# Protocol for a meta-review of interventions to prevent and manage ICU delirium

**DOI:** 10.1136/bmjopen-2024-090815

**Published:** 2025-02-11

**Authors:** Katherine Louise Jones, Burak Kundakci, Andrew Booth, Maria Pufulete, Ben Gibbison

**Affiliations:** 1 The University of Sheffield, Sheffield, England, UK; 2 University of Bristol, Bristol, UK; 3 Department of Cardiac Anaesthesia and Intensive Care, University Hospitals Bristol and Weston NHS Foundation Trust, Bristol, UK

**Keywords:** Delirium, Adult intensive & critical care, Review, Patient Care Management, THERAPEUTICS

## Abstract

**Abstract:**

**Introduction:**

Intensive care unit (ICU) delirium is an acute brain dysfunction that affects up to 7 out of 10 patients admitted to ICUs. Patients who develop ICU delirium cannot think clearly, have trouble paying attention, do not understand what is happening around them and may see or hear things that are not there. ICU delirium increases the time patients spend in ICUs and hospitals and therefore healthcare costs. ICU delirium is also associated with increased mortality and dementia in the longer term. ICU delirium prevention and management strategies are likely to include both pharmacological and non-pharmacological components as part of a complex intervention, but it is unclear which components should be included. The objective of this meta-review is to systematically map the quantity and certainty of the available evidence from reviews and meta-analyses of randomised controlled trials (RCTs) of pharmacological and non-pharmacological interventions, which will be used to design a multicomponent intervention to prevent and manage ICU delirium.

**Methods and analysis:**

A systematic search strategy was performed in MEDLINE (Ovid), Embase (Elsevier), Cochrane Database of Systematic Reviews, Scopus, Cumulative Index to Nursing and Allied Health Literature (CINAHL), PsycINFO and Web of Science (from inception to 26 September 2023), as well as Epistemonikos (from inception to 19 July 2023). We will include all critically ill adults (aged≥18 years) and any ICU delirium prevention or management intervention (pharmacological or non-pharmacological). For pharmacological interventions, we will include reviews of RCTs. For non-pharmacological interventions, we will consider reviews of RCTs, quasi-experimental and cohort studies. We will use the International Consensus Study (Del-COrS) core outcome set for research evaluating interventions to prevent or manage ICU delirium and synthesise our findings using quantitative data description methods. We will involve our Patient and Public Involvement group of people who experienced ICU delirium to develop and comment on such aspects as the research question, methodology and which outcomes are most important.

**Ethics and dissemination:**

No ethical approval is required for this study. The results of this meta-review will be disseminated through peer-reviewed publications and conferences. They will also form part of an evidence map and logic model for the prevention and management of ICU delirium.

**PROSPERO registration number:**

CRD42023473260

**STRENGTHS AND LIMITATIONS OF THIS STUDY**

- This systematic meta-review will provide a comprehensive overview of the evidence and evidence gaps pertaining to interventions to prevent and manage intensive care unit (ICU) delirium.
- The meta-review will include interventions to prevent and manage ICU delirium, but determine the effect on each separately.
- The meta-review will help to identify potential limitations contributing to the complexity of evidence synthesis and implementation research in ICU delirium.
- We will limit the meta-review to English-language-only publications, which could miss relevant evidence.
- The meta-review will not include qualitative evidence, which we will explore separately in future work.

## Introduction

### Description of the condition

 Intensive care unit (ICU) delirium is an acute brain dysfunction that affects up to 7 out of 10 patients admitted to intensive care.^1^ In the UK, this equates to over 171 000 patients developing ICU delirium in intensive care each year, although current diagnostic tools are suboptimal and may underestimate the true extent of ICU delirium. This number is set to increase as more older people and people with comorbidities are admitted to intensive care.

Patients who develop ICU delirium cannot think clearly, have trouble paying attention, do not understand what is happening around them and may see or hear things that are not there. This is extremely distressing for both patients and their families. Many factors contribute to the likelihood of developing delirium, including the illness that leads to the ICU admission, comorbidities and the medications that are used in ICU (eg, sedatives and analgesia), infections, severe pain, the brain’s inability to use oxygen and withdrawal from alcohol and nicotine.

There are three broad, clinical manifestations of ICU delirium; hyperactive, hypoactive or mixed, affecting<2%, 45% and 53%^2 3^ of patients, respectively. Patients with hyperactive delirium are aggressive and restless and may interrupt their treatment by pulling out invasive catheters and ventilation equipment. Patients with hypoactive delirium are inattentive, non-engaged and stuporous. In mixed delirium, patients fluctuate between hyperactive and hypoactive delirium.

### Why it is important to do this review

ICU delirium increases the time patients spend in intensive care and in hospital (HR for discharge 0.65, 95% CI 0.55 to 0.76)^4^ and therefore healthcare costs (by around £13 000 per stay).^5 6^ ICU delirium is also associated with increased mortality^4 7^ and dementia^8 9^ in the longer term. Assessing patients for delirium was an unmet part of the Dementia 2020 Challenge of the UK Department of Health and Social Care,^10^ listed as high-priority research by National Institute for Health and Care Excellence/Royal College of Physicians^11^ and is in the top three priorities of the James Lind Alliance’s Intensive Care Priority Setting Partnership.^12^ It is therefore a shared priority for clinicians, patients and family members and healthcare decision-makers to prevent ICU delirium and shorten its duration when it develops.

Both pharmacological and non-pharmacological interventions have been used to prevent and manage ICU delirium. Pharmacological interventions may include avoidance of benzodiazepines, use of dexmedetomidine for sedation,^13^ antipsychotics^14^ and melatonin.^15^ Non-pharmacological interventions may include repeated reorientation of patients, mobilisation, sleep protocols and use of a scheduled pain management tool. Individual interventions have been tested in numerous randomised controlled trials (RCTs). The optimal intervention is expected to include multiple components, although these have not been adequately defined and agreed upon by clinicians. The ABCDEF bundle^16^ developed and promoted by the US Society of Critical Care Medicine is one example of a defined complex intervention. It has been found to improve mortality, ICU and hospital stays,^17^ but barriers to its implementation include, for example, increased workload and lack of clinician engagement because of perceived lack of efficacy.^18^ Some aspects of the ABCDEF bundle are difficult to apply to the UK setting due to the differences in ICU organisation, staffing structure and case mix between the USA and UK.^19 20^

RCTs of single pharmacological interventions have been combined in multiple systematic reviews, meta-analyses and network meta-analyses. However, there has been no overarching review of the evidence base that also explores the conduct and reporting of findings, with potential implications for practice and research design, as well as the methodological expectations for reviews of ICU delirium.

## Methods and analysis

### Aim

To provide an overview of the evidence from systematic reviews of pharmacological and non-pharmacological interventions to prevent and manage ICU delirium.

### Objectives

1. To identify systematic reviews of RCTs that involve single or combination pharmacological interventions or sedation protocols to prevent or manage ICU delirium.
2. To identify systematic reviews of RCTs or quasi-experimental and cohort studies that involve single or combination non-pharmacological interventions (with or without pharmacological components) to prevent or manage ICU delirium.
3. To synthesise systematic review findings against an established minimum core outcome set, as well as other important outcome measures, and assess review conduct and reporting.
4. To create an evidence map to understand the extent of the evidence on interventions for ICU delirium.

We will apply systematic evidence synthesis methods to the conduct of a meta-review (review of reviews and umbrella review), in alignment with guidance to produce Cochrane overviews.^21^ Reporting for the protocol follows the Preferred Reporting Items for Systematic review and Meta-Analysis (PRISMA) Protocols 2015 checklist^22^ and is available separately (online supplemental file 1).

### Patient and public involvement statement

For the duration of the review process, we will involve our Patient and Public Involvement group of people who have lived experience of ICU delirium to help develop and comment on such aspects as the research question, methodology and which outcomes are most important from the patient and carer perspective.

### Types of reviews

We will include all published systematic reviews in English with or without meta-analyses from 2000 to the present day. Intensive care has changed significantly since the year 2000. The number of ICU beds has increased,^23^ the staffing and technology have improved and intensive care is now a stand-alone specialty in the UK and internationally,^24^ with its own faculty, training programme and governance structures.^25^ Included primary studies published pre-2000 and post-2000 will be recorded and discussed.

We will include reviews regardless of which country the primary research was conducted in. For pharmacological interventions we will include reviews of RCTs, since the RCT evidence is known to be extensive. Reviews of pharmacological interventions with mixed study designs including RCTs will only be included if separate analyses are reported for RCTs. For non-pharmacological interventions, we will also consider quasi-experimental and cohort studies if there are no relevant RCTs for an intervention. We will include scoping and mapping reviews but exclude narrative reviews without systematic searches, protocols, abstract-only citations and reviews not published in the English language. Integrative reviews will be considered if they have relevant included study designs. Any overviews of reviews will be recorded but excluded from data extraction and evidence mapping.

### Types of participants

We will include all critically ill adults (aged≥18 years). We define critically ill patients as those treated in a critical care or ICU of any specialty (eg, burn, cardiac, medical, surgical and trauma) or high dependency unit. Reviews of postoperative delirium will only be considered for inclusion if they relate to the ICU setting. Reviews focused on ICU subpopulations, such as post-surgery or those receiving mechanical ventilation, will be considered as subgroups within the meta-review. We will exclude those studies conducted in other intermediate care units (eg, coronary care units, respiratory high care units). Potentially relevant reviews of mixed settings including ICU (eg, general hospital ward and ICU) will be identified and findings synthesised and mapped only if≥80% of included studies are reported to be conducted in the ICU. We will exclude studies of delirium related to alcohol withdrawal.

### Types of interventions

Any delirium prevention, treatment or management intervention (pharmacological or non-pharmacological). This may include single interventions, care packages/bundles or services interventions that are compared with either another intervention, a placebo, no treatment and standard or usual care. We will include deprescribing as an intervention (eg, spontaneous awakening trials and avoidance of benzodiazepines).^26^ Similar interventions may be used to prevent and treat/manage delirium once it has occurred (to shorten its duration). Because we want to identify all relevant evidence, we will include all approaches and distinguish between reviews of preventative interventions and treatment or management interventions. Reviews that do not specify intervention as prophylactic, treatment or management for ICU delirium will be included, and this uncertainty will be recorded.

### Outcomes

We will use the Del-COrS core outcome set for research evaluating interventions to prevent or manage delirium in critically ill adults.^27^ We will assess the outcomes separately for interventions designed to (1) prevent and (2) treat or manage delirium.

- **Prevent:primaryoutcome**

Delirium occurrence: defined as either prevalence (the number of new and/or existing cases during the reporting period) or incidence (new cases that occur during the reporting period). Although most RCTs of delirium prevention interventions are expected to use the term ‘delirium incidence’ as their primary outcome (the intuitive endpoint of a preventive intervention), delirium occurrence is considered more appropriate because it is difficult to establish exactly when delirium starts in any given patient. Many patients arrive in intensive care asleep or heavily sedated and current delirium diagnostic tools are unable to assess delirium unless patients are awake even though delirium may already have started. Also, delirium fluctuates (may get better, then get worse again, then get better and so on) and it is difficult to capture the first episode.

Delirium occurrence, prevalence or incidence may be used interchangeably in the literature, so for the purpose of our meta-review, all will be classed as delirium occurrence. Where reviews report outcomes separately for occurrence, incidence and prevalence, this will be recorded.

**Prevent:secondary outcomes**

1. ICU length of stay (days or hours as reported by the review).
2. Hospital length of stay (days or hours as reported by the review).
3. Mortality (at any time point reported by the review).
4. Time to delirium resolution or duration of delirium (at any time point reported by the review).
5. Delirium severity (measured using any scale and timing, as reported by the review).
6. Change in cognition including memory (measured using any cognitive scale and timing, as reported by the review).
7. Change in emotional distress including anxiety, depression, acute stress or post-traumatic stress disorder* (using any symptom screening scale or diagnostic criteria at any time point reported by the review).
8. Change in health-related quality of life (using any scale at any time point reported by the review).

- **Treat or manage:primary outcomes**

Time to delirium resolution or duration of delirium (days or hours as reported by the review) or delirium recurrence.

**Treat or manage:secondary outcomes**

1. ICU length of stay (days or hours as reported by the review).
2. Hospital length of stay (days or hours as reported by the review).
3. Mortality (at any time point reported by the review).
4. Delirium severity (measured using any scale and timing, as reported by the review).
5. Change in cognition including memory (measured using any cognitive scale and timing, as reported by the review).
6. Change in emotional distress including anxiety, depression, acute stress or post-traumatic stress disorder* (using any symptom screening scale or diagnostic criteria at any time point reported by the review).
7. Change in health-related quality of life (using any scale at any time point reported by the review).

*Post-traumatic stress disorder as a new diagnosis involves the presence of symptoms for at least 1 month but will be extracted where reported.

### Search strategy

A systematic search strategy was performed in MEDLINE (Ovid), Embase (Elsevier), Cochrane Database of Systematic Reviews, Scopus, Cumulative Index to Nursing and Allied Health Literature (CINAHL), PsycINFO and Web of Science (from inception to 26 September 2023), as well as Epistemonikos (from inception to 19 July 2023). The search strategy was developed and run by experienced Information Specialists (including AB) in collaboration with the review team. Our MEDLINE (Ovid) search strategy is available in online supplemental file 2.

### Selection of reviews

This meta-review involves a team of professionals with expertise across health services research and clinical medicine. We imported search results into Excel for screening. Two of three reviewers (of KLJ, BK and AB) independently screened the search results at title/abstract followed by full text with the removal of duplicate records. Dual, independent screening was completed against meta-review eligibility criteria for a subset of at least 20% of records at title/abstract and full text. Screening results were then aggregated by one reviewer (AB).

We will undertake further screening of all included full texts as part of data extraction by at least two of three reviewers (of KLJ, BK and AB). Any disagreements will be resolved through consensus discussion with the review team and any full texts unavailable during screening will be recorded. We will review the reference lists of excluded overviews of reviews for additional reviews not found by our initial search. We will describe included and excluded studies within a PRISMA style flow chart during the various stages of the review and explain our reasons for excluding reviews.

### Data extraction

We will perform data extraction using a standardised data extraction form in Excel, developed by the review authors and pilot‐tested on at least five systematic reviews to ensure it captures all relevant data. Reviewers will extract data including the author, dates of publication (year), publication title / publishing journal, publication study design characteristics, details about the population, sample size, interventions, comparisons, outcomes (including composite delirium outcomes, eg, delirium-free and coma-free days) and any reported concurrent interventions not included in the comparison. Different doses and modes of intervention delivery will be extracted where reported. We will also extract information of effect or association and adverse events of the intervention, including on specific subgroups, if reported. We will (where possible, if included in the review) extract information from the review about the tools or instruments used to diagnose delirium and psychiatric diagnoses. Classification of intervention as prevention, treatment or management will be based on review author reporting.

We will identify tools or instruments used to appraise the strength of the evidence from primary research included in systematic reviews (eg, Cochrane tool for risk of bias versions 1 and 2, Jadad scale, Newcastle-Ottawa scale or its adapted version) and any further investigations of the risk of bias through funnel plots and sensitivity analysis. Use of random or fixed effects models and I^2^ assessment of heterogeneity in meta-analyses (0–100%, whereby 0–40% might not be important, 30–60% moderate heterogeneity, 50–90% substantial heterogeneity and 75–100% considerable heterogeneity^28^) will be extracted where reported. We will extract GRADE assessments of the certainty of the evidence where available. Guidance has been developed to support the consistency of such assessments in overviews, however, further assessment will not be performed for evidence mapping.^29^

### Unit of analysis

The unit of analysis is the systematic review. A potential source of unit of analysis issues within systematic reviews of ICU delirium will be the meta-analysis of cluster and individual RCTs together. We will extract both types of RCTs from systematic reviews and, where reported, how they were handled in meta-analysis. Cross-over RCTs are not anticipated to be a research design applied to the meta-review population. The handling of multiple arm studies and multiple observations for the same outcome may be further sources of unit of analysis issues, for example, repeated measurements and recurrence of ICU delirium. Definitions of outcomes will be extracted where reported, including the time point of measurement, as well as any individual patient data meta-analysis.

### Data synthesis

We will narratively synthesise and report quantitative findings from included reviews. We will present findings according to PICOTS (Population / Patients, Intervention, Comparator, Outcome, Time, Setting) reported in the included evidence. Formal meta-analysis is not planned for evidence mapping although documentation of quantitative effect sizes will be used to highlight further specific opportunities for meta-analysis and broader implications for future research. Evidence syntheses that involve network meta-analysis or pairwise meta-analysis will be presented separately with discussion of the consistency of findings.

We will begin by mapping the types of interventions included in the evidence base, the overlap of included studies and summary characteristics of included reviews. This initial mapping will help to identify review comprehensiveness and inform the development of the synthesis strategy. Subsequently, we will create evidence tables and summaries of evidence to provide detailed overviews of the included systematic reviews and their findings.

We will create evidence maps based on the included review PICOTS and the type of evidence synthesis. Separate maps are proposed for pharmacological and non-pharmacological interventions, particularly important given the different evidence thresholds being applied.

### Reporting

We aim to apply approaches taken for Cochrane overviews,^21^ the Preferred Reporting Items for Overviews of Reviews (PRIOR)^30^ and PRISMA extension for Scoping Reviews^31^ to guide and inform the reporting of this meta-review. Any deviations from the protocol will be recorded as part of the meta-review.

## Discussion

This systematic meta-review will provide an overview and map of the evidence pertaining to interventions to prevent and/or manage delirium in the ICU. This map, in conjunction with surveys and qualitative research in ICUs across the UK, will inform future research to establish ‘the best way to tackle ICU delirium in the UK’.

There are many systematic reviews of interventions to prevent and manage ICU delirium. There are also two overviews including systematic reviews^32 33^ that focus on pharmacological or non-pharmacological interventions separately and prophylaxis across different hospital settings. This review is the first systematic meta-review that focuses on the ICU setting and aims to describe both pharmacological and non-pharmacological evidence relevant to intensive care, producing translational clinical evidence maps.

The strengths of the review methodology are that it has been designed with a multidisciplinary team of clinicians, methodologists and patients to provide a set of outcomes that are considered core for research and which answer important questions regarding outcomes for patients. It complements other work packages within our programme of work, designed to provide a comprehensive picture of how patient-centred ICU delirium research should progress within the UK. Our meta-review will help to identify potential limitations contributing to the complexity of evidence synthesis and implementation research in ICU delirium. The review methodology has some weaknesses. For example, included evidence will be limited to English language publications due to logistical constraints, which could miss some relevant evidence in other languages. Also, we will not include qualitative evidence in our meta-review although this will be explored separately in future work.

### Ethics and dissemination

No ethical approval is required for this study, and patient consent for publication is not applicable. The results of this meta-review will be disseminated through peer-reviewed publications and conferences. They will also form part of an evidence map and logic model for the prevention and management of ICU delirium.

## supplementary material

10.1136/bmjopen-2024-090815 online supplemental file 1

10.1136/bmjopen-2024-090815 online supplemental file 2
