# Supplementary material for: Protocol for a meta-review of interventions to prevent and manage ICU delirium
Source: BMJ Open. 2025 Feb 11;15(2):e090815. doi: 10.1136/bmjopen-2024-090815 (PMC11815468; doi:10.1136/bmjopen-2024-090815)
Supplement: online supplemental file 1 [file bmjopen-15-2-s001.docx]

**Supplement 2. Search strategy**

**MEDLINE (Ovid)**

and Ovid MEDLINE(R) In-Process & Other Non-Indexed Citations $$ Medline (Dates searched: 1946 to September 26, 2023). Number of references retrieved: 528.

**1** exp confusion/
**2** deliri*.ti,ab.
**3** (acute adj2 (confusion* or "brain syndrome" or "brain failure" or "psycho-organic syndrome" or "organic psychosyndrome" or "organic brain syndrome")).ti,ab.
**4** (terminal* adj restless*).ti,ab.
**5** (toxic adj2 (confus$ or psychosis)).ti,ab.
**6** metabolic encephalopathy.ti,ab.
**7** clouded state.ti,ab.
**8** "clouding of consciousness".ti,ab.
**9** exogenous psychosis.ti,ab.
**10** or/1-9
**11** exp Intensive Care Units/
**12** Intensive Care.ti,ab.
**13** ICU.ti,ab.
**14** Critical care/
**15** (Critical adj2 (care or ill or illness*)).ti,ab.
**16** (high dependency unit* or HDU).ti,ab.
**17** or/11-16
**18** (systematic review or meta-analysis).pt.
**19** meta-analysis/ or systematic review/ or systematic reviews as topic/ or meta-analysis as topic/ or exp technology assessment, biomedical/ or network meta-analysis/
**20** ((systematic* adj3 (review* or overview*)) or (methodologic* adj3 (review* or overview*))).ti,ab,kf. (331263)
**21** ((quantitative adj3 (review* or overview* or synthes*)) or (research adj3 (integrati* or overview*))).ti,ab,kf.
**22** ((integrative adj3 (review* or overview*)) or (collaborative adj3 (review* or overview*)) or (pool* adj3 analy*)).ti,ab,kf.
**23** (data synthes* or data extraction* or data abstraction*).ti,ab,kf.
**24** (handsearch* or hand search*).ti,ab,kf.
**25** (mantel haenszel or peto or der simonian or dersimonian or fixed effect* or latin square*).ti,ab,kf.
**26** (met analy* or metanaly* or technology assessment* or HTA or HTAs or technology overview* or technology appraisal*).ti,ab,kf.
**27** (meta regression* or metaregression*).ti,ab,kf.
**28** (meta-analy* or metaanaly* or systematic review* or biomedical technology assessment* or bio-medical technology assessment*).mp,hw.
**29** (medline or cochrane or pubmed or medlars or embase or cinahl).ti,ab,hw.
**30** (cochrane or (health adj2 technology assessment) or evidence report).jw.
**31** (comparative adj3 (efficacy or effectiveness)).ti,ab,kf.
**32** (outcomes research or relative effectiveness).ti,ab,kf.
**33** ((indirect or indirect treatment or mixed-treatment or bayesian) adj3 comparison*).ti,ab,kf.
**34** (multi* adj3 treatment adj3 comparison*).ti,ab,kf.
**35** (mixed adj3 treatment adj3 (meta-analy* or metaanaly*)).ti,ab,kf.
**36** umbrella review*.ti,ab,kf.
**37** (multi* adj2 paramet* adj2 evidence adj2 synthesis).ti,ab,kf.
**38** (multiparamet* adj2 evidence adj2 synthesis).ti,ab,kf.
**39** (multi-paramet* adj2 evidence adj2 synthesis).ti,ab,kf.
**40** or/18-39
**41** and/10,17,40

*Lines 18 to 39 are taken from the [CADTH SR/MA/HTA/ITC - MEDLINE, Embase, PsycInfo](https://searchfilters.cadth.ca/list?q=&ps=20&topic_facet=health%20technology%20assessments%20000000%7CHealth%20technology%20assessments&p=1&name_facet=medline%20000000%7CMEDLINE) search filter, adapted for Ovid Medline.
