# Supplementary material for: Protocol for a meta-review of interventions to prevent and manage ICU delirium
Source: BMJ Open. 2025 Feb 11;15(2):e090815. doi: 10.1136/bmjopen-2024-090815 (PMC11815468; doi:10.1136/bmjopen-2024-090815)
Supplement: Supplementary file 3 [file bmjopen-2024-090815.reviewer_comments.pdf]

PEER REVIEW HISTORY

BMJ Open publishes all reviews undertaken for accepted manuscripts. Reviewers are asked to complete a checklist review form and are provided with free text boxes to elaborate on their assessment. These free text comments are reproduced below.

ARTICLE DETAILS

Title (Provisional)

Protocol for a meta-review of interventions to prevent and manage ICU Delirium

Authors

Jones, Katherine Louise; Kundakci, Burak; Booth, Andrew; Pufulete, Maria; Gibbison, Ben

---

VERSION 1 - REVIEW

**Reviewer**	**1**
**Name**	**Potter, Kelly M**
**Affiliation**	**University of Pittsburgh**
**Date**	**12-Aug-2024**
**COI**	**n/a**

Thank you for this important work to systematically map the quantity and certainty of evidence for pharmacological and non-pharmacological interventions to prevent or treat ICU delirium. I have a few minor comments that will improve the publication of this protocol:

- ICUD is not a standard abbreviation in delirium, and may confuse readers. Please revise to ICU delirium.

- P4L17: The psychomotor subtypes of delirium (hyperactive, hypoactive, and mixed delirium) are just one mechanism of classifying delirium, as others have been published more recently (clinical phenotypes of delirium, data-driven subtypes of delirium). Please either expand your discussion of "types" of delirium to include other typologies, or specify that you describe subtypes of symptom presentation.

- P4L20: "Quietly confused" is not an accurate description of hypoactive delirium, and ignores inattention which has been a defining feature of hypoactive delirium. Please revise your description of hypoactive delirium.

- P4L36-37: "Pharmacological interventions may include avoidance of benzodiazepines" - Is de-prescribing really a pharmacological intervention? You later (P4L39) describe spontaneous awakening trials as a non-pharmacological intervention. Please revise for clarity.

- P4L39-40: Mobility is also an important non-pharmacological intervention to mention in your description.

-P5L40-41: Will you include integrative reviews? These are also systematically conducted and guided by an established framework. (PMID 16268861)

-P6L22-25: How will you handle composite outcomes (e.g., delirium- and coma-free days)?

VERSION 1 - AUTHOR RESPONSE

Thank you and Dr Kelly Potter for this helpful feedback on our protocol manuscript. Please find our responses to peer review below, and our revised copies of the manuscript (marked and clean) attached.

1. ICUD is not a standard abbreviation in delirium, and may confuse readers. Please revise to ICU delirium.
Thank you. We have amended our reporting throughout the manuscript accordingly.
2. P4L17: The psychomotor subtypes of delirium (hyperactive, hypoactive, and mixed delirium) are just one mechanism of classifying delirium, as others have been published more recently (clinical phenotypes of delirium, data-driven subtypes of delirium). Please either expand your discussion of "types" of delirium to include other typologies, or specify that you describe subtypes of symptom presentation.
Thank you. We included these as a description that clinicians would recognise and to show that “hypoactive” delirium is prevalent, even though it often does not pose an obvious clinical problem to clinicians (aggressive “hyperactive” people do). A discussion of the subtypes of delirium in the ICU is beyond the scope of this article. However, we have clarified within the text that these are broad clinically seen types.
3. P4L20: "Quietly confused" is not an accurate description of hypoactive delirium, and ignores inattention which has been a defining feature of hypoactive delirium. Please revise your description of hypoactive delirium.
Thank you. We have changed the wording of this within the Introduction to “Patients with hypoactive delirium are inattentive, non-engaged and stuporous.”
4. P4L36-37: "Pharmacological interventions may include avoidance of benzodiazepines" - Is de-prescribing really a pharmacological intervention? You later (P4L39) describe spontaneous awakening trials as a non-pharmacological intervention. Please revise for clarity.
Thank you. We have added a new reference to support our decision to include deprescribing as part of the prescribing process and a pharmacological intervention (Langford et al., 2024). Spontaneous awakening trials classify as deprescribing and so we have amended our reporting of this to pharmacological intervention, thank you.
5. P4L39-40: Mobility is also an important non-pharmacological intervention to mention in your description.
Thank you, we agree this is an important intervention to mention and have added this into the Introduction.
6. P5L40-41: Will you include integrative reviews? These are also systematically conducted and guided by an established framework. (PMID 16268861)
Thank you. We have updated the Methods and analysis section to clarify that "Integrative reviews will be considered if they have relevant included study designs."
7. P6L22-25: How will you handle composite outcomes (e.g., delirium- and coma-free days)?
Thank you. We have updated the Data extraction subsection of the Methods and analysis to clarify that we will extract "...outcomes (including composite delirium outcomes e.g.,

delirium- and coma-free days)" although our meta-review focuses on the Del-COrS core-outcome set.

We also made three additional edits to (i) correct the final reference listed for an existing, non-pharmacological overview from Kim et al., 2018 to Souza, Azzolin and Fernandes, 2018 (ii) correct the reference format for Page et al., 2021, and (iii) remove reference to the version of general software used in the Methods and analysis section.
